# West Nile Virus from Blood Donors, Vertebrates, and Mosquitoes, Puerto Rico, 2007

**DOI:** 10.3201/eid1508.090333

**Published:** 2009-08

**Authors:** Elizabeth A. Hunsperger, Kate L. McElroy, Kovi Bessoff, Candimar Colón, Roberto Barrera, Jorge L. Muñoz-Jordán

**Affiliations:** Centers for Disease Control and Prevention, San Juan, Puerto Rico, USA

**Keywords:** West Nile virus, Caribbean, surveillance, Puerto Rico, blood donors, falcon, horse, viruses, mosquitoes, dispatch

## Abstract

West Nile virus (WNV) was isolated from a human blood donor, a dead falcon, and mosquitoes in Puerto Rico in 2007. Phylogenetic analysis of the 4 isolates suggests a recent introduction of lineage I WNV that is closely related to WNV currently circulating in North America.

The first human cases of West Nile virus (WNV) in the Caribbean were documented in the Cayman Islands and the Florida Keys in 2001 (*1*). Antibody-positive animals were described in 2002 in Guadeloupe, the Dominican Republic, Jamaica, and eastern Mexico (*2**–**4*). Most of the sequencing information and phylogenetic analysis are primarily on isolates from the United States. Viral isolates from outside the United States were obtained from Mexico in 2003 and Argentina in 2006 (*2**,**5*). Although many studies have described seropositive animals in the Americas and the Caribbean, isolates were not commonly obtained for virus characterization.

The presence of WNV antibodies in nonmigratory birds was first reported in Puerto Rico in 2004 (*6*). In response to this discovery, serum samples from 345 healthy, unvaccinated horses and 14 dead horses were tested by plaque reduction neutralization test (PRNT) during 2004–2005. Three horses from Fajardo municipality were confirmed seropositive for WNV by PRNT. Additionally, samples from 408 free-ranging chickens were tested by hemagglutination inhibition assay and two chickens from Arecibo municipality were seropositive. However, the sample volumes were insufficient for confirmation by PRNT (Centers for Disease Control and Prevention [CDC], unpub. data). During 2004–2006, no additional WNV-positive samples were detected. We hypothesized that the virus could have been circulating on the island without causing detectable illness or death in humans, horses, or birds.

## The Study

**Figure 1:**
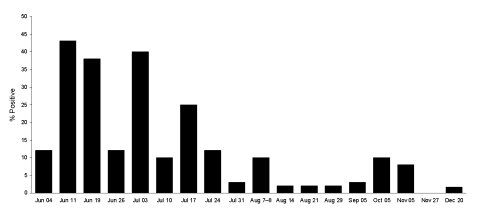
Summary of surveillance data indicating zoonotic and human transmission of West Nile virus (WNV) in Puerto Rico in 2007. Percentage of anti-WNV immunoglobulin (Ig) M–positive chickens per week from June 4 through December 20. Chickens were bled weekly during the beginning of transmission and monthly starting in September 2007. Sixty chickens were placed in the sentinel surveillance sites as previously described by Barrera et al. (*7*).

To test this hypothesis, we selected a study site in Ceiba and Naguabo municipalities, where the first birds positive for WNV were detected (*6**,**7*). Sixty sentinel chickens were placed in groups of 5 in 12 locations in accordance with Institutional Animal Care and Use Committee policies as previously described (*7*). The first seroconversion occurred in June 2007, one year after the initiation of the surveillance. Up to 43% of the chickens seroconverted in June and July 2007, as detected by immunoglobulin M ELISA (*8*); the percentage declined during September through December (Figure 1). During the period of transmission, miniature light/CO_2_ traps and gravid traps from CDC were placed near the chicken cages to capture mosquitoes. Samples from pools of *Culex nigripalpus*, *Cx. bahamensis*, and *Cx. quinquefasciatus* mosquitoes were positive for WNV RNA, as detected by real-time reverse transcription–PCR (RT-PCR) (*7*). Sixteen WNV isolates were obtained by inoculating either C6/36 (*Aedes albopictus*) or Vero (African green monkey kidney) cell cultures with mosquito pool extract.

In addition to the study site, WNV activity was detected throughout the island from June through mid-September 2007. In June 2007, WNV in 3 human blood donations from San Juan, Gurabo, and Vega Baja municipalities were detected by the American Red Cross (ARC) screening. Two blood donation samples were positive for WNV by RT-PCR, and 1 sample yielded an isolate of WNV confirmed by immunofluorescence (*9*). In September 2007, an encephalitic horse died in Cabo Rojo municipality and a dead falcon (Falco sparverius*)* was reported from the neighboring Mayaguez municipality. Brain tissue from the horse and falcon were WNV positive by RT-PCR, and an isolate was obtained from the falcon. Neurologic symptoms were reported in 3 other horses in these municipalities, but no specimens were available for testing.

**Figure 2:**
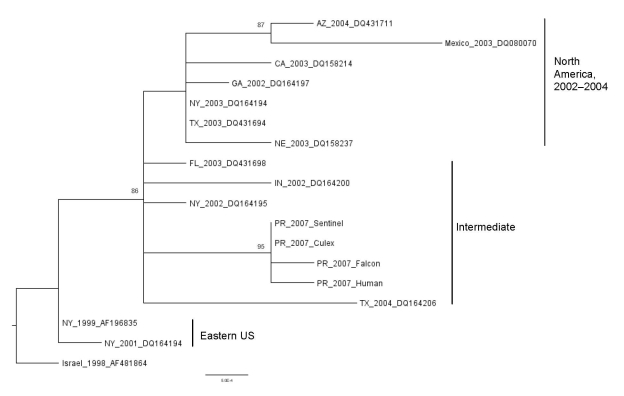
Identification of West Nile virus (WNV) in Puerto Rico. WNV premembrane-envelope maximum likelihood phylogenetic tree of WNV isolates collected from 1998 through 2007, demonstrating the relationship of viruses from Puerto Rico to other lineage I isolates. Viruses are labeled by place and year of isolation and GenBank accession no. FJ799714–FJ799717 (Fiji). Clade names are consistent with those used by Davis et al. (*14*). Numbers indicate the neighbor-joining bootstrap values for groups in the tree and are shown when >70. Scale bar indicates nucleotide substitutions per site.

WNV isolates from the falcon, human blood donation, sentinel chicken (*7*), and a pool of *Cx. nigripalpus* mosquitoes were sequenced to assess their phylogenetic relationships (Figure 2). Viral RNA was extracted from first passage Vero cell supernatant using the QIAamp Viral RNA kit (QIAGEN, Valencia, CA, USA). RT-PCR was performed by using the OneStep RT-PCR kit (QIAGEN) with WNV-specific primers (*9*), and DNA bands were excised and purified using the QIAquick gel-extraction kit (QIAGEN). The 2,004-nt premembrane-envelope (prM-E) genes of both DNA strands were directly sequenced by using the BigDye Terminator v3.1 Cycle Sequencing kit on a 3130X Genetic Analyzer (Applied Biosystems, Foster City, CA, USA).

These newly obtained WNV sequences were aligned with other WNV sequences from GenBank database by ClustalW in MEGA (www.megasoftware.net) (*10**,**11*). Neighbor-joining (NJ) and minimum-evolution analyses were also performed in MEGA. Maximum-likelihood (ML) analysis was performed by using PAUP* (*12*) under the best-fit TrN model selected by Modeltest 3.7 (*13*). Bootstrapping was performed (1,000 replications) by the NJ method using the substitution model specified for the ML analysis. The ML phylogeny analysis is depicted in Figure 2. On the basis of previous work, most North American WNV isolates fall into 3 clades: Eastern United States (including NY99); North American, 2002–2004 (the dominant clade); and an intermediate clade. All phylogenetic analyses yielded similar tree topologies with high (95%–96%) bootstrap support for placement of the Puerto Rican WNV isolates into a monophyletic group within the intermediate clade identified by Davis et al. (*14*).

Within the West Nile virus Puerto Rico (WNV_PR) group, the isolates from all 4 sources were identical at the amino acid level, and only 2-nt differences were observed, prM-A114G in PR_2007_Falcon and E-C276T in PR_2007_Human. PR_2007_Sentinel and PR_2007_*Culex* isolates were identical at the nucleotide level. The WNV-PR viruses differed from the consensus of the other sequences analyzed at 3 nucleotides: E-T201C, E-G537A, and E-A561G. None of these substitutions resulted in an aa difference. Consistent with a previous report (*7*), all WNV-PR isolates contained the valine to alanine substitution at the E-159 characteristic of WNV isolated in North America after 2001. Overall sequence homology of WNV-PR viruses to NY99 was 99.7% nt and 99.8% aa.

## Conclusions

The chicken surveillance in Puerto Rico demonstrated active WNV transmission in the Caribbean and a pattern of transmission with peak activity occurring from early June through late July in 2007 and decreasing from August through December 2007. The peak WNV activity in chickens coincided with the human, falcon, and horse infections. Although blood donations to the ARC from Puerto Rico had been consistently screened for WNV since June 2003 (≈80,000 donations/year), WNV was first detected in 3 donations in June 2007 <10 miles from one another and >40 miles east of the sentinel chicken site (*15*). Moreover, the WNV-infected falcon and horse died on the western region of the island in September. Together, these findings support seasonality in WNV transmission in Puerto Rico. Alternatively, WNV might be reintroduced to Puerto Rico sporadically with long periods of low or no transmission, which could explain the apparent absence of zoonotic or human transmission from the end of 2004 through May 2007.

To assess the relationship of WNV_PR with other WNV strains, we sequenced isolates obtained from the sentinel chicken, human, falcon, and *Culex* spp. mosquito pool. The 2,004-nt prM-E region was sufficient based on previous evidence to produce an accurate phylogeny (*14*). The high sequence homology between PR_2007 viruses was expected, given that previous studies found little genetic difference in strains isolated in the same period and geographic space (*14*). Our analysis suggests the introduction of a single clade of WNV in Puerto Rico before June 2007. Unfortunately, determining the exact origin of the PR_2007 isolates was not possible in this analysis.

Active transmission of WNV in Puerto Rico indicated that this virus has established a life cycle of natural, amplifying hosts and arthropod vectors with horses and humans as dead-end hosts. Sequence analysis of 4 isolates indicates that PR_WNV was derived from a recent introduction of lineage I WNV and is closely related to WNV currently circulating in North America based on the location of the isolates in the phylogenetic tree. Additional WNV isolates from Puerto Rico are necessary to assess the impact of WNV on the ecology and human disease in the tropics.
